# Aesthetic correction of lesion by post-liposuction corticoid infiltration using subcision, PMMA filling, and CO_2_ laser

**DOI:** 10.1080/23320885.2019.1602837

**Published:** 2019-04-23

**Authors:** Roberto Chacur, Honório Sampaio Menezes, Nívea Maria Bordin da Silva Chacur, Danuza Dias Alves, Rodrigo Cadore Mafaldo, Leandro Dias Gomes, Gina Matzenbacher, Gisele dos Santos Barreto

**Affiliations:** Leger Clinic, Rio de Janeiro, Brazil

**Keywords:** Lipectomy, pigmentation, polymethyl methacrylate, lasers, gas, injectable corticosteroids

## Abstract

Introduction: Post-liposuction fibrosis is a relatively common complication which can be repaired. Case report: We report a case of cutaneous atrophy and diffuse irregularity of the abdominal region with achromia post-liposuction. She was treated with subcision, PMMA filler and fractioned CO_2_ laser. Results: Cutaneous colour and irregularities get improved.

## Introduction

The liposuction technique has evolved considerably in many countries, being one of the most popular aesthetic procedures for sculpting the human body [1–3]. It promotes the removal of subcutaneous fat, thus, correcting body lipodystrophies. For faster recovery time, shorter hospital stays, and less complicated post-operative periods, while considering smaller areas, local anaesthesia is indicated. For larger areas, the Klein’s tumescent technique or epidural analgesia, with or without sedation, are suggested [4]. Some local postoperative complications may occur, such as seromas, haematomas, skin irregularities (visible and palpable), fibrosis, necrosis, and scarring. Systemic complications, such as visceral perforations, allergic reactions to intra- and postoperative medications, fever, systemic infections, fatty embolism, sepsis, and death may also occur [4].

Human skin after a tissue injury may respond with the formation of fibrosis [5], which is the main sequelae related to the scarring process in the postoperative period. Collagen is a protein found abundantly in the human body. Its development in excess or its accumulation during tissue repair generates fibrosis [5,6]. Injectable corticosteroids, which is used to inhibit the production of collagen, acts as an inhibitor of alpha-2 macroglobulin. This inhibits the action of collagenase type V, which promotes a decrease in the action of fibroblasts. Such inhibition controls the fibrotic scar process, being also used to control gynoid lipodystrophy. The use of injectable corticosteroids has been an option for the treatment of fibrosis. Nevertheless, the physician carrying out the treatment should be aware of the possible side effects related to different prescribed forms and doses [7]. Some complications are due to intralesional injection, such as the development of telangiectasias, cutaneous atrophy, and hypo- or hyperpigmentation of the skin [8].

We present a case report with corrective treatment for severe cutaneous atrophy caused by injectable triamcinolone used to improve post-liposuction fibrosis.

## Case report

**Figure 1.**
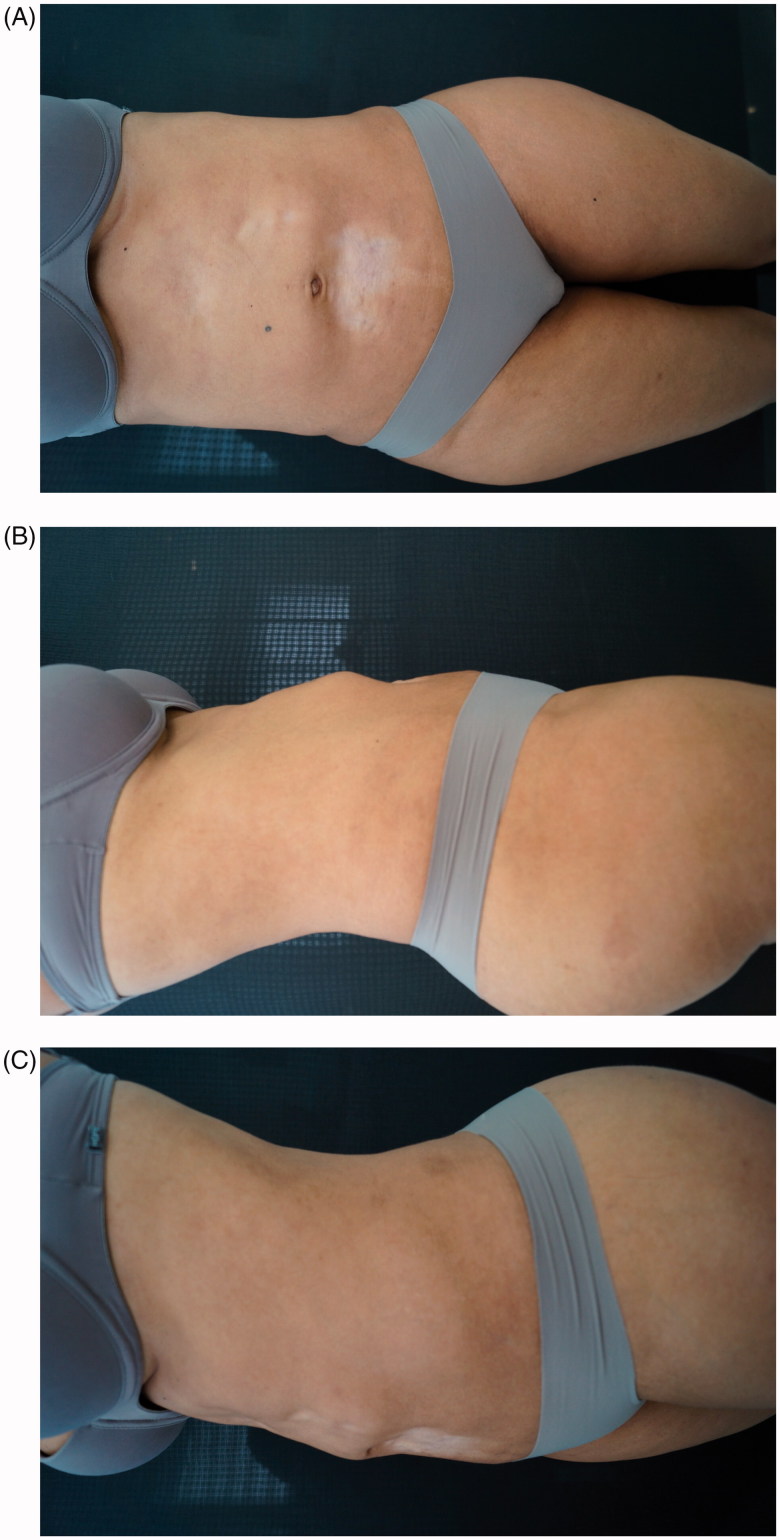
(A,B,C) Before treatment.

A 40-year-old woman, from San Paulo, presented with severe cutaneous atrophy, achromia and irregular skin texture due to the attempt to correct abdominal post-liposuction fibrosis by using corticosteroid infiltration. Two years before, the patient had undergone an abdominal liposuction procedure, followed by local application of triamcinolone, in an attempt to correct permanent fibrosis resulting from the liposuction. Injecting triamcinolone (unknown dose), aggravated the cutaneous atrophy, abdominal irregularity, and achromia conditions (Figure 1).

This case report was approved by the Research Ethics Committee of the Universidade Veiga de Almeida (UVA/RJ) (CAAE protocol number 97197618.4.0000.5291).

There is no conflict of interest.

**Figure 2.**
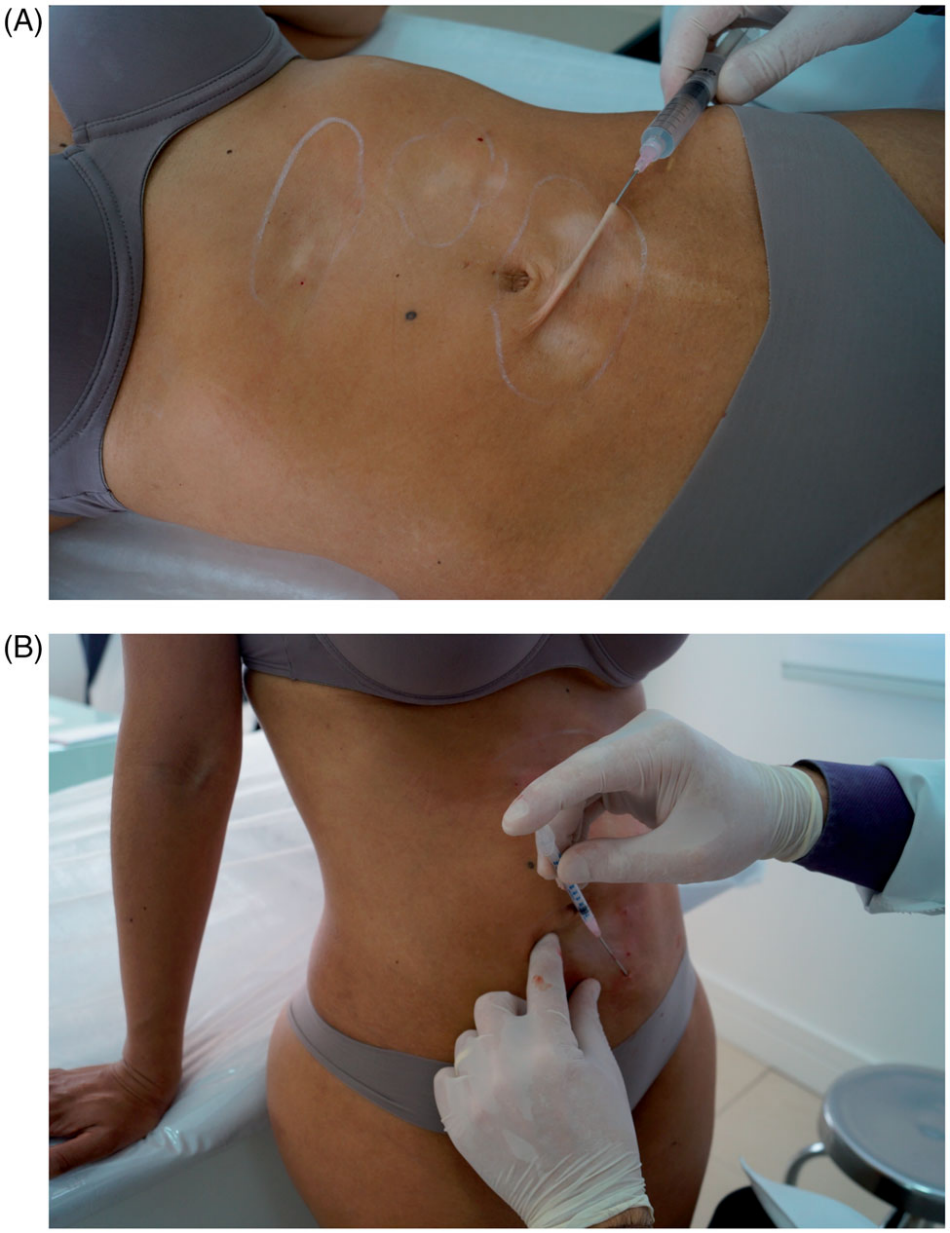
(A-B) Treatment fill with synthetic implant.

**Figure 3.**
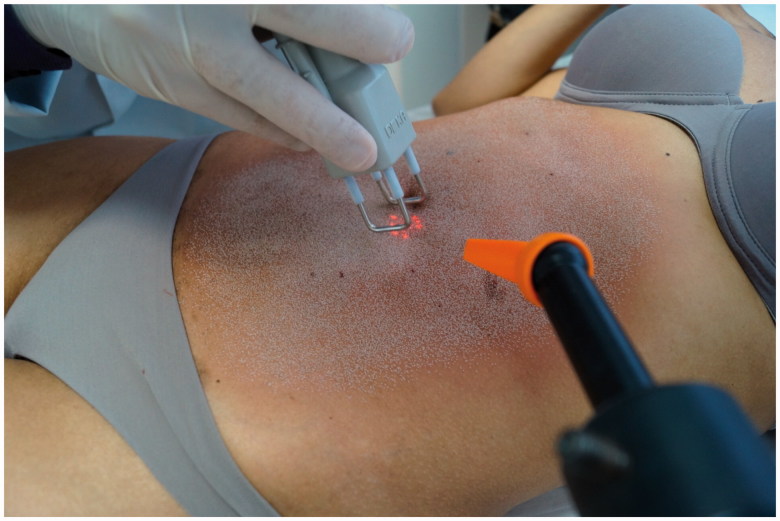
Treatment with fractional CO_2_ laser.

**Figure 4.**
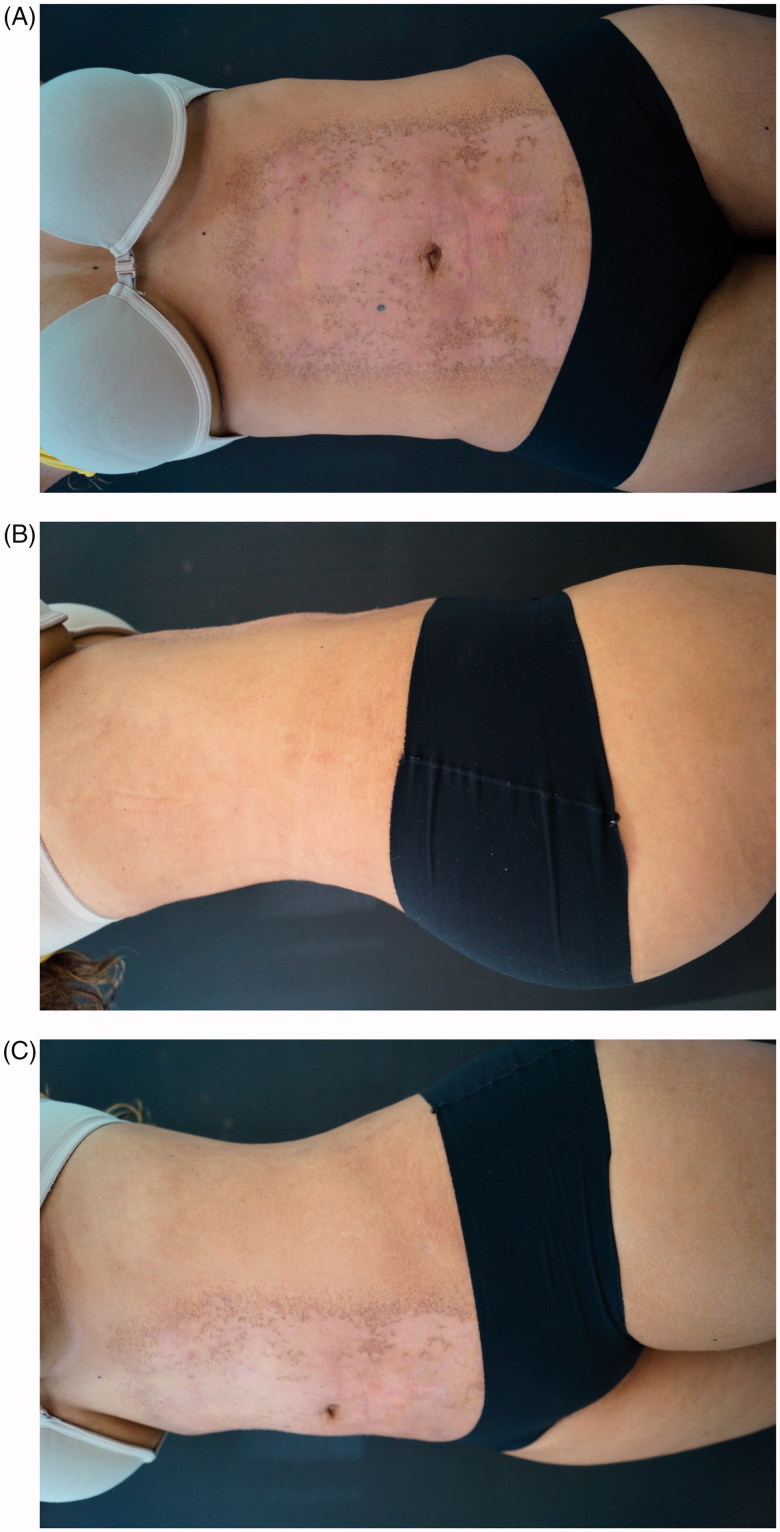
(A,B,C) During treatment.

Subcision treatment was carried out in a single session. During this session, 26 ml of polymethyl methacrylate 10% were implanted with a micro-canula (Figure 2(A,B)). A CO_2_ laser with radiofrequency coupled was applied soon after filling (Figures 3 and 4).

## Results

**Figure 5.**
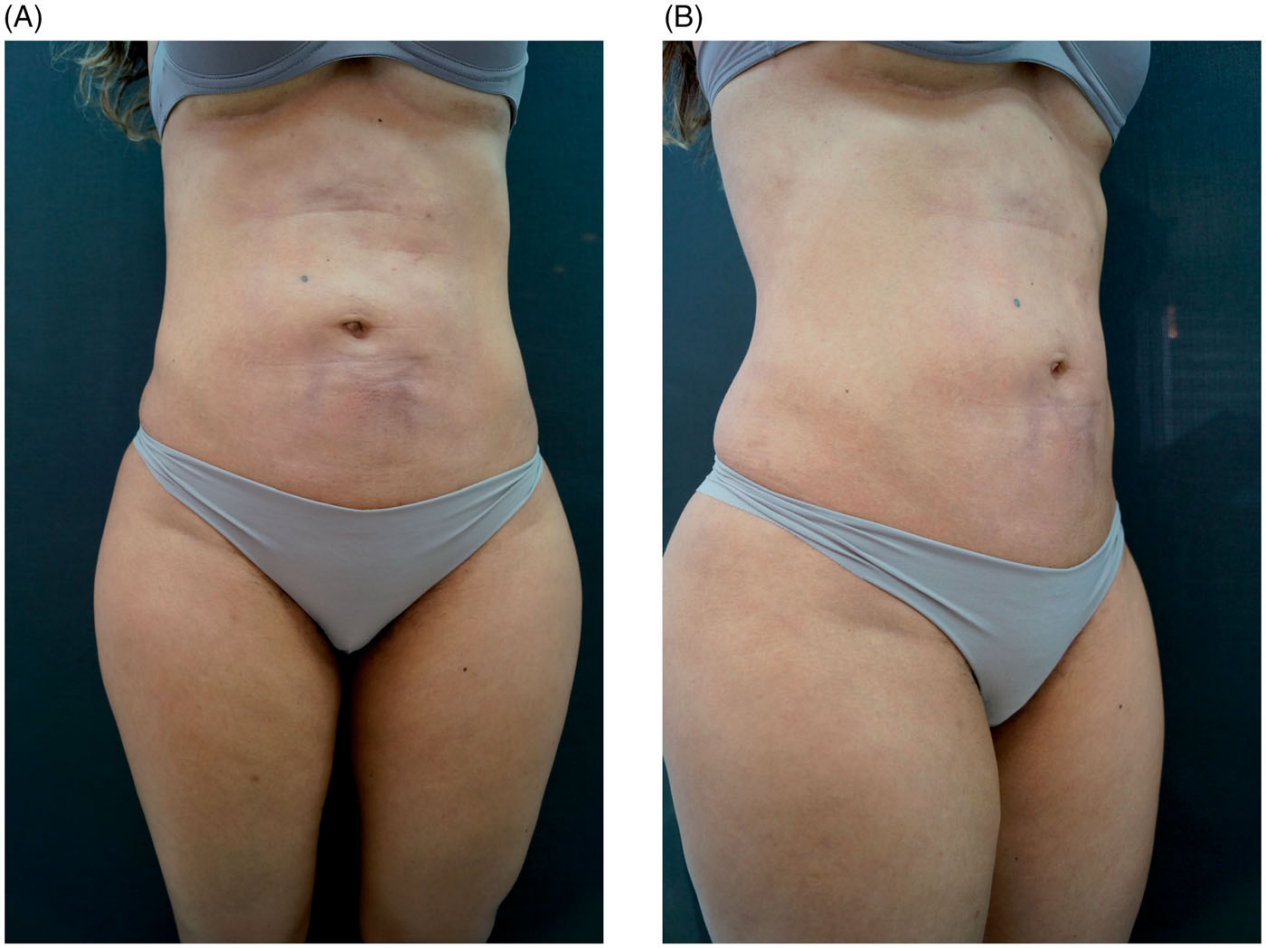
(A,B) One month after treatment.

**Figure 6.**
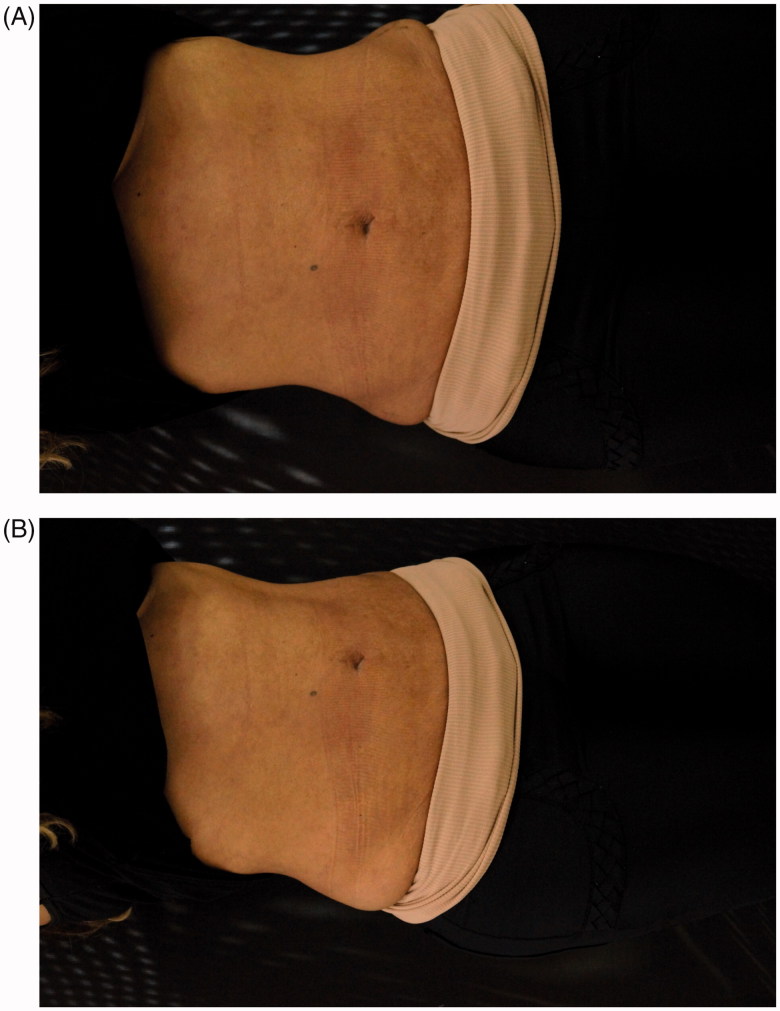
(A,B) 4-years follow-up treatment.

One month later we noted improvement of skin appearance (Figure 5). Four years follow-up demonstrated very satisfactory results (Figure 6).

## Discussion

In this case, the treatment of fibrosis and its adhesions, tissue atrophy, and colour homogenisation had the objective of correcting the aesthetic damage, correction which was sought with the treatments applied to the patient. The use of injectable intralesional corticosteroids may cause some deformities in the skin [8], as presented in this case.

Injectable corticosteroid, used to inhibit the production of collagen, is known to act by inhibiting alpha-2 macroglobulin. This inhibits the action of collagenase type V, which promotes a decrease in the action of fibroblasts, thus, controlling the scar process [8].

Subcision was used to correct the skin relief by means of a Y-shaped instrument, sliding it back and forth in a fanlike movement, thus dissecting the fibrotic tissue until the skin is released [9].

Subsequently, tissues were filled with PMMA, a product which is non-absorbable by the human body. Its microspheres are phagocytosed by macrophages, the vehicle being degraded slowly and replaced by new collagen produced from the inflammatory process, stimulating the formation of 80% of connective tissue, which will remain in place as long as the particles are present [10]. Neocollagenesis, which results from the stimulation of the fibroblast caused by the inflammatory process using PMMA (in this case beneficial), has an exact reverse action of the injectable corticosteroid. Thus, it allows the correction of the tissue atrophy in a relatively easy way, with a procedure that can be performed in the doctor’s office, using local anaesthesia, and presenting permanent effect. When applied superficially these products need to be more diluted or well distributed to prevent the formation of nodules or irregularities. PMMA, when the abdomen is considered, is indicated for treatments of depressed scars, of stretch marks when there is excessive depression, and of correction and depression in the abdominal fat layer after liposuction [11].

The fat grafting procedure option was not given due the fact that should be performed in a surgical block, under anaesthesia. A lack of predictable volume retention is clearly a characteristic of autologous fat grafting which would only temporarily increase the volume, would not stimulate the production of new collagen and would not lead to the same desired result as with pmma filling [12].

Subcutaneous filling with PMMA is also widely used in patients with HIV/AIDS associated Lipodystrophy Syndrome [13,14] as well as in Romberg hemifacial atrophy, reconstructing lost angles and contours due to volume reduction by fat reabsorption (lipoatrophy). PMMA was the best choice for the case because it is a non-toxic material, which does not induce hypersensitivity or foreign body reactions, which does not degenerate over time or induce calcification, because it is chemically inert and easily implantable. In addition to the cost of the treatment being accessible to the patient.

For the correction or improvement of dermal and subdermal skin irregularities, it is possible to use radiofrequency, which was, indeed, carried out with the patient, since the CO_2_ laser device had a radiofrequency head attached to it. According to Atiyeh and Dibo, improvement in the irregularities of deep dermis can be observed after the use of radiofrequency. The thermal effects of radiofrequency promote the denaturation of collagen, with immediate contraction of its fibres and subsequent activation of the fibroblasts with neocolagenesis of the collagen fibres over time, due to a secondary healing response and subsequent remodelling of the cutaneous tissue [15,16].

The CO_2_ laser is a major advance on existing conventional methods to ablate the epidermis, thereby inducing regrowth of a young-looking epidermis and stimulating collagenesis and remodelling in the dermis, which stimulate its healing response. Fractional lasers create microscopic heat columns causing areas of thermal damage known as microscopic thermal zones. The denaturation of the collagen takes place due to the heat emitted by the laser, causing the fibres to shrink quickly, and hardening the skin [17]. The collagenase levels rise and the healing of the lesion that degrades the collagen matrix begins [18]. Therefore, the CO_2_ laser from adjacent cells reconstitutes both the dermis and the epidermis [19].

The treatment proposed in this work aimed to correct the cicatricial adhesions and to the repigment the skin. A significant improvement was observed using four techniques together. The subdermal PMMA filling, which was used to stimulated the production of collagen (inhibited by the corticosteroid), to restore the volume, and to correct the irregularities of the skin, presented good results, just as the Radiofrequency correction of subdermal skin irregularities and fractionated CO_2_ laser epidermic repigmentation.

## Conclusion

The association of subcision, filling with polymethyl methacrylate, and CO_2_ laser with radiofrequency produced satisfactory treatment of skin deformity after corticosteroid administration in abdominal liposuction surgery.
